# Ischemic preconditioning attenuates ischemia/reperfusion-induced kidney injury by activating autophagy via the SGK1 signaling pathway

**DOI:** 10.1038/s41419-018-0358-7

**Published:** 2018-03-01

**Authors:** Ying Xie, Daofang Jiang, Jing Xiao, Chensheng Fu, Zhenxing Zhang, Zhibin Ye, Xiaoli Zhang

**Affiliations:** 1 Department of Nephrology, Huadong Hospital, Fudan University, Shanghai, China; 2 Shanghai Key Laboratory of Clinical Geriatric Medicine, Shanghai, China

## Abstract

Ischemic preconditioning (IPC) has a strong renoprotective effect during renal ischemia/reperfusion (I/R) injury that is thought to relate to autophagy. However, the role of autophagy during IPC-afforded renoprotection and the precise mechanisms involved are unknown. In this study, an in vitro hypoxia/reoxygenation (H/R) model was established in which oxygen and glucose deprivation (OGD) was applied to renal cells for 15 h followed by reoxygenation under normal conditions for 30 min, 2 h or 6 h; transient OGD and subsequent reoxygenation were implemented before prolonged H/R injury to achieve hypoxic preconditioning (HPC). 3-Methyladenine (3-MA) was used to inhibit autophagy. In a renal I/R injury model, rats were subjected to 40 min of renal ischemia followed by 6 h, 12 h or 24 h of reperfusion. IPC was produced by four cycles of ischemia (8 min each) followed by 5 min of reperfusion prior to sustained ischemia. We found that IPC increased LC3II and Beclin-1 levels and decreased SQSTM/p62 and cleaved caspase-3 levels in a time-dependent manner during renal I/R injury, as well as increased the number of intracellular double-membrane vesicles in injured renal cells. IPC-induced renal protection was efficiently attenuated by pretreatment with 5 mM 3-MA. Pretreatment with IPC also dynamically affected the expression of SGK1/FOXO3a/HIF-1α signaling components. Moreover, knocking down SGK1 expression significantly downregulated phosphorylated-FOXO3a (p-FOXO3a)/FOXO3 and HIF-1α, suppressed LC3II and Beclin-1 levels, increased SQSTM/p62 and cleaved caspase-3 levels, and abolished the protective effect of IPC against I/R-induced renal damage. SGK1 overexpression efficiently increased p-FOXO3a/FOXO3 and HIF-1α levels, promoted the autophagy flux and enhanced the protective effect mediated by HPC. Furthermore, FOXO3a overexpression decreased HIF-1α protein levels, inhibited HIF-1α transcriptional activity and reduced the protective effect of IPC. Our study indicates that IPC can ameliorate renal I/R injury by promoting autophagy through the SGK1 pathway.

## Introduction

Acute kidney injury (AKI) is a severe clinical syndrome and a major contributor to morbidity and mortality^1^. Ischemia/reperfusion (I/R) injury is a common cause of AKI in patients experiencing acute stress, such as surgery, organ transplantation, trauma, sepsis, or shock^2^. Ischemic preconditioning (IPC), which comprises short sublethal cycles of I/R prior to the prolonged ischemic insult, provides protection against renal I/R injury^3,4^. IPC has been shown to have a strong renoprotection effect due to its anti-apoptosis/necrosis, anti-inflammatory and anti-oxidant properties^5^. Autophagy is a highly conserved lysosome-dependent degradation pathway for recycling damaged cytosolic proteins and organelles to facilitate cellular homeostasis and promote cell survival under stress conditions, such as nutrient deprivation and hypoxia^6^. The induction of autophagy was recently shown to attenuate renal I/R injury^1,2^. However, the role of autophagy during IPC-afforded renoprotection and the precise mechanisms involved are poorly understood.

Hypoxia inducible factor-1 (HIF-1) is an oxygen-sensitive transcription factor composed of an O_2_-regulated HIF-1α subunit and a constitutively expressed HIF-1β subunit. HIF-1α is continuously synthesized and targeted for ubiquitin-mediated degradation by HIF prolyl hydroxylase (PHD) under normoxic conditions; under hypoxic conditions, HIF-1α degradation is inhibited, leading to protein accumulation and dimerization with HIF-1β, thus facilitating the transcription of numerous genes involved in the adaptive cellular and systemic responses to oxygen deprivation^7^. HIF activation might be particularly beneficial in I/R. Our previous study demonstrated that HIF-1 activation by pretreatment with dimethyloxalylglycine, a PHD inhibitor, induced delayed renal protection against I/R injury in mice^8^. Importantly, emerging evidence has implicated HIFs in the process of IPC^9,10^. Yamanaka-Tatematsu et al.^11^ reported that autophagy activated by cobalt chloride, which induces HIF-1α overexpression, is an energy source that maintains homeostasis and plays a protective role against prolonged hypoxia. Based on these evidences, we speculated that HIF-1α-mediated autophagy plays an important role in IPC.

Serum and glucocorticoid induced kinase-1 (SGK1) is a serine/threonine kinase initially identified as a gene transcriptionally regulated by glucocorticoids and serum^12^. The expression and activation of SGK1 were later shown to be regulated by various cell stress stimuli, such as hyperosmotic stress, ultraviolet radiation, heat shock, oxidative stress, and hypoxia^13^. Under these conditions, SGK1 acts as a strong anti-apoptotic kinase. Previous studies showed that SGK1 overexpression blunts the apoptotic insult in renal cells exposed to hypoxia/reoxygenation (H/R)^14,15^. Brunet et al.^16^ demonstrated that the SGK1/forkhead transcription factor 3a (FOXO3a) pathway promotes cell survival by inhibiting cell cycle arrest and apoptosis. Importantly, a previous study reported that HIF-1 transcriptional activity can be negatively regulated by FOXO3a^17^. Considering above evidences, we wondered whether the SGK1/FOXO3a/HIF-1α signaling pathway is involved in the protection afforded by IPC.

In this study, we intended to investigate whether IPC regulates autophagy in renal cells exposed to I/R injury and exerts renoprotection through the SGK1/FOXO3a/HIF-1α signaling pathway.

## Results

### Hypoxic preconditioning alleviates hypoxia/reoxygenation-induced renal tubular epithelial cell damage by inducing autophagy in vitro

**Fig. 1:**
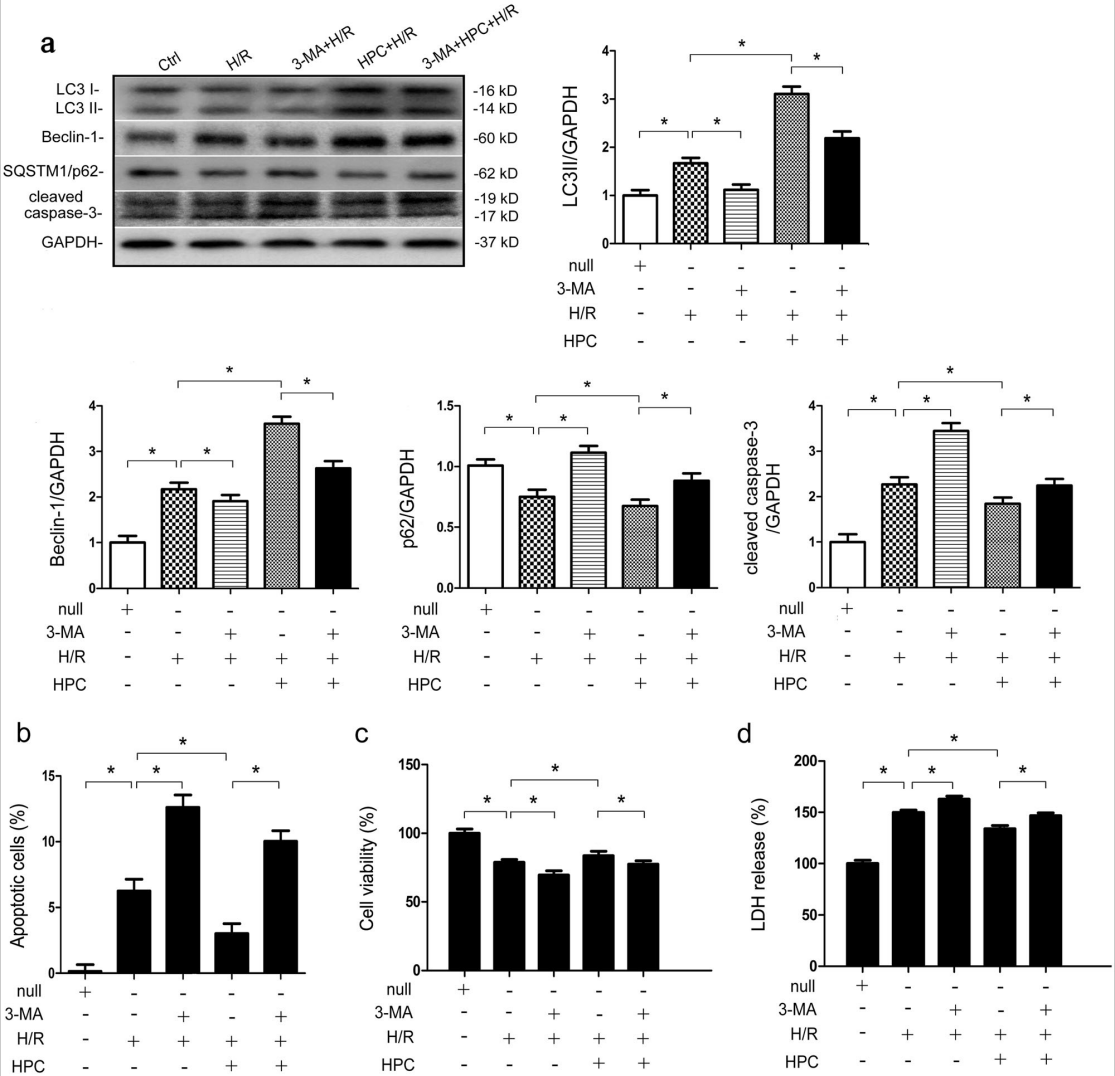
Hypoxic preconditioning (HPC) protects renal tubular cells from hypoxia/reperfusion (H/R) injury by inducing autophagy in vitro. **a** HK-2 cells were cultured under normal or oxygen and glucose deprivation (OGD) conditions for 15 h, followed by reoxygenation in normal medium for 2 h with or without prior HPC, which consisted of OGD for 6 h and subsequent reoxygenation for 2 h. The inhibitor 3-methyladenine (3-MA; 5 mM) was added to the medium 1 h before each experiment to suppress autophagy. Western blotting and subsequent data analysis were performed to ascertain LC3II, Beclin-1, SQSTM1/p62, and cleaved caspase-3 protein levels in HK-2 cells. Representative data are presented. **b** Determination and quantitative analysis of apoptotic cells by Annexin V-propidium iodide FACS analysis. **c**, **d** Renal tubular cell viability and lactic dehydrogenase (LDH) release were evaluated by CCK-8 and LDH assays. Each experiment was conducted at least three times independently. The data are presented as the mean ± S.D. *n* = 3^*^.*P* < 0.05

Besides morphological observation, the primary method for measuring autophagic activity involves the detection of Beclin-1, SQSTM1/p62 and LC3I to LC3II processing. As shown in Fig. 1a, intracellular Beclin-1, LC3II, and cleaved caspase-3 were increased after H/R injury compared with control (*P* < 0.05 vs. Ctrl). The protein levels of SQSTM1/p62, a selective substrate of autophagy, were depleted (*P* < 0.05 vs. Ctrl). After a 1-h incubation with 5 mM 3-methyladenine (3-MA), cleaved caspase-3 levels in HK-2 cells were further increased after H/R injury (*P < *0.05 vs. H/R). Increases in LC3II, Beclin-1 and decreases in SQSTM1/p62, cleaved caspase-3 were evident in the HPC+H/R group compared with the H/R group (*P < *0.05 vs. H/R) whereas these changes were abolished by 3-MA (*P < *0.05 vs. HPC+H/R). Apoptosis was then measured by flow cytometric analysis. As shown in Fig. 1b, H/R resulted in an increased percentage of apoptotic cells (H/R 6.26 ± 0.89% vs. Ctrl 0.16 ± 0.5%; *P < *0.05), which was suppressed by HPC (HPC+H/R 3.02 ± 0.75%; *P* < 0.05 vs. H/R). Regardless of HPC pretreatment, 3-MA treatment increased apoptosis after H/R injury (3-MA + H/R 12.6% ± 0.96% vs. H/R; 3-MA + HPC + H/R 10.04% ± 0.8% vs. HPC + H/R; *P < *0.05).CCK-8 and LDH assays showed that HPC increased HK-2 cells viability after H/R injury (*P* < 0.05 vs. H/R) (Fig. 1c, d), but this protective effect was abolished by 3-MA (*P* < 0.05 vs. HPC + H/R), indicating that the inhibition of autophagy is correlated with increased apoptosis and cell injury. HPC is involved in the protective effect against renal H/R injury in an autophagy-dependent manner.

### SGK1 promotes renal protection by hypoxic preconditioning during hypoxia/reoxygenation injury in vitro via the FOXO3a/HIF-1α signaling pathway

**Fig. 2:**
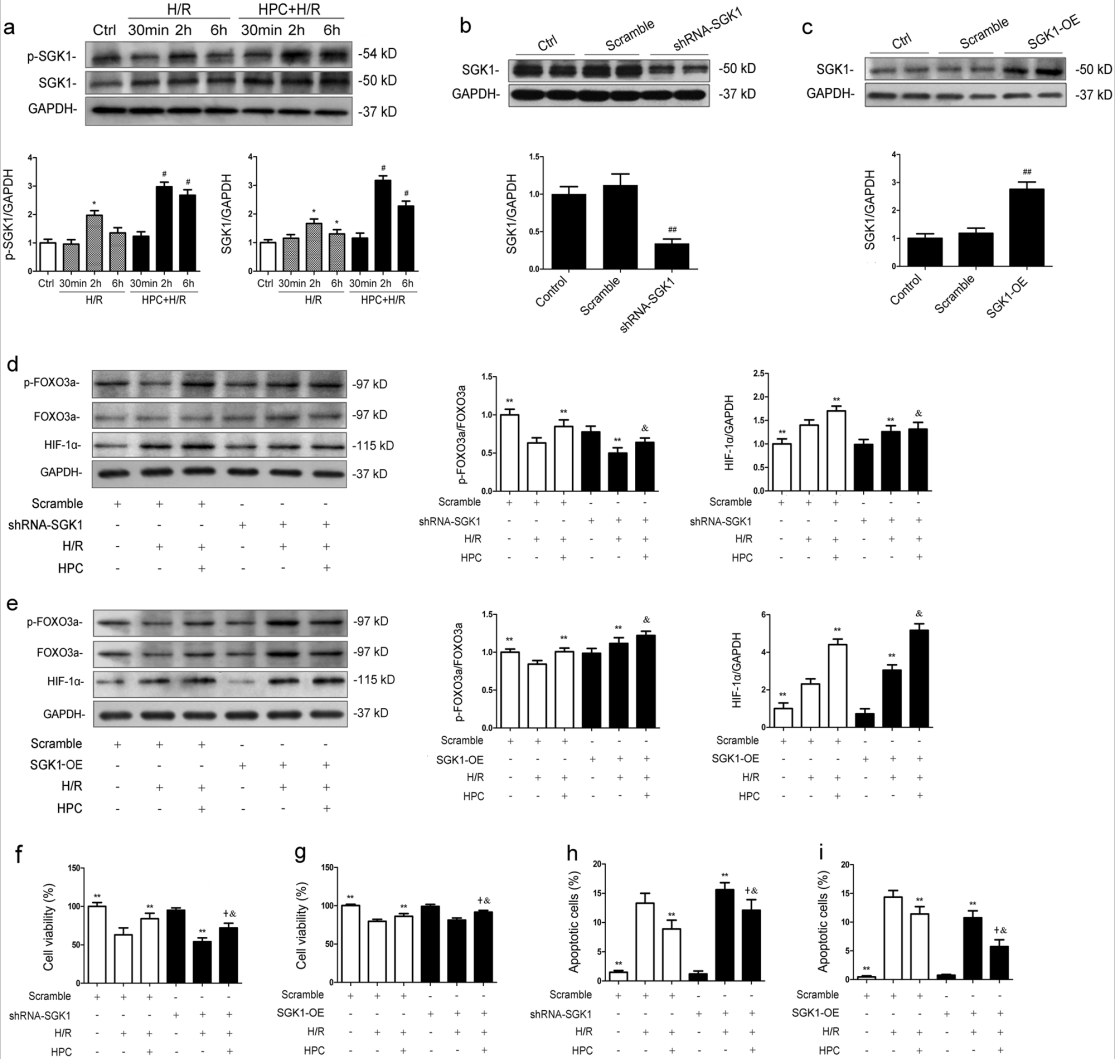
The SGK1/FOXO3a/HIF-1α signaling pathway is involved in HPC-mediated renal protection during H/R injury in vitro. **a** HK-2 cells were cultured under normal or oxygen and glucose deprivation (OGD) conditions for 15 h, followed by reoxygenation in normal medium for 30 min, 2 h or 6 h with or without prior HPC, which consisted of OGD for 6 h and subsequent reoxygenation for 2 h. p-SGK1 and SGK1 protein levels were evaluated by western blotting. **b**, **c** Western blotting analysis of SGK1 knockdown and overexpression efficacy. Cells were transfected with Scramble, shRNA-SGK1 or pEX-3-SGK1. After 48 h, total protein was extracted for western blotting analysis. **d**, **e** Cells were transfected with Scramble, shRNA-SGK1 or pEX-3-SGK1. After 48 h, they were cultured under normal conditions or exposed to H/R injury with or without prior HPC. After reoxygenation for 2 h, cells were collected for western blotting detection of p-FOXO3a, FOXO3a and HIF-1α. Representative data are presented. **f**, **g** Renal tubular cell viability was evaluated by CCK-8 assay. **h**, **i** Flow cytometry was used to analyze the percentage of apoptotic cells. All the data are derived from an experiment that was repeated at least three times. The data are presented as the mean ± S.D. *n* = 3^*^.*P* < 0.05 vs. Control; ^#^*P* < 0.05 vs. H/R group at the corresponding reoxygenation time; ^##^*P* < 0.05 vs. Scramble; ^**^P < 0.05 vs. Scramble + H/R; ^&^*P* < 0.05 vs. Scramble + HPC + H/R; ^+^*P* < 0.05 vs. shRNA-SGK1 + H/R or SGK1-OE + H/R

SGK1 and p-SGK1 levels were increased at 2 h after reoxygenation compared with the control (*P* < 0.05 vs. Ctrl) (Fig. 2a). The levels of SGK1 and p-SGK1 were not different between HPC + H/R-30 min and H/R-30 min groups but were significantly increased by pretreatment with HPC at H/R-2 h and H/R-6 h (HPC+H/R-2 h vs. H/R-2 h; HPC + H/R-6 h vs. H/R-6 h; *P* < 0.05). Then we used RNA interference and full-length cDNA transfection to knockdown or upregulate intracellular SGK1 expression, respectively. Efficient SGK1 silencing and overexpression were detected in cells transfected with shRNA-SGK1 (*P* < 0.05 vs. Scramble) (Fig. 2b) and pEX-3-SGK1 (*P* < 0.05 vs. Scramble, Fig. 2c). P-FOXO3a/FOXO3a level was decreased while HIF-1α protein level was increased after H/R injury (*P* < 0.05 vs. Scramble); HPC significantly increased p-FOXO3a/FOXO3a and HIF-1α protein levels after H/R injury (*P* < 0.05 vs. Scramble + H/R) (Fig. 2d, e). Knocking down SGK1 markedly reduced p-FOXO3a/FOXO3a and HIF-1α expressions in the HPC + H/R group (*P* < 0.05 vs. Scramble + HPC + H/R) (Fig. 2d), while overexpression of SGK1 enhanced p-FOXO3a/FOXO3a and HIF-1α expressions in the HPC + H/R group (*P* < 0.05 vs. Scramble + HPC + H/R) (Fig. 2e). CCK-8 assays showed that HPC restored cell viability after H/R injury (*P* < 0.05 vs. Scramble + H/R) (Fig. 2f, g). However, SGK1 knockdown with shRNA-SGK1 before H/R exposure significantly attenuated HPC-conferred promotion of cell viability (*P* < 0.05 vs. Scramble + HPC + H/R) (Fig. 2f), and SGK1 overexpression with pEX-3-SGK1 promoted HPC-conferred protection of cell viability (*P* < 0.05 vs. Scramble + HPC + H/R) (Fig. 2g), suggesting the ability of SGK1 to promote cell viability during H/R. Moreover, pretreatment with HPC significantly decreased the percentage of apoptotic cells induced by H/R injury (*P* < 0.05 vs. Scramble + H/R) (Fig. 2h, i), and the increase in apoptotic cells after shRNA-SGK1 transfection (*P* < 0.05 vs. Scramble + HPC + H/R) (Fig. 2h) and the decrease in them after pEX-3-SGK1 transfection (*P* < 0.05 vs. Scramble + HPC + H/R) (Fig. 2i) both indicated that HPC alleviated H/R-induced renal cell apoptosis by increasing SGK1 expression.

### FOXO3a affects HIF-1α signaling pathway and hypoxic preconditioning-conferred renoprotection in vitro

**Fig. 3:**
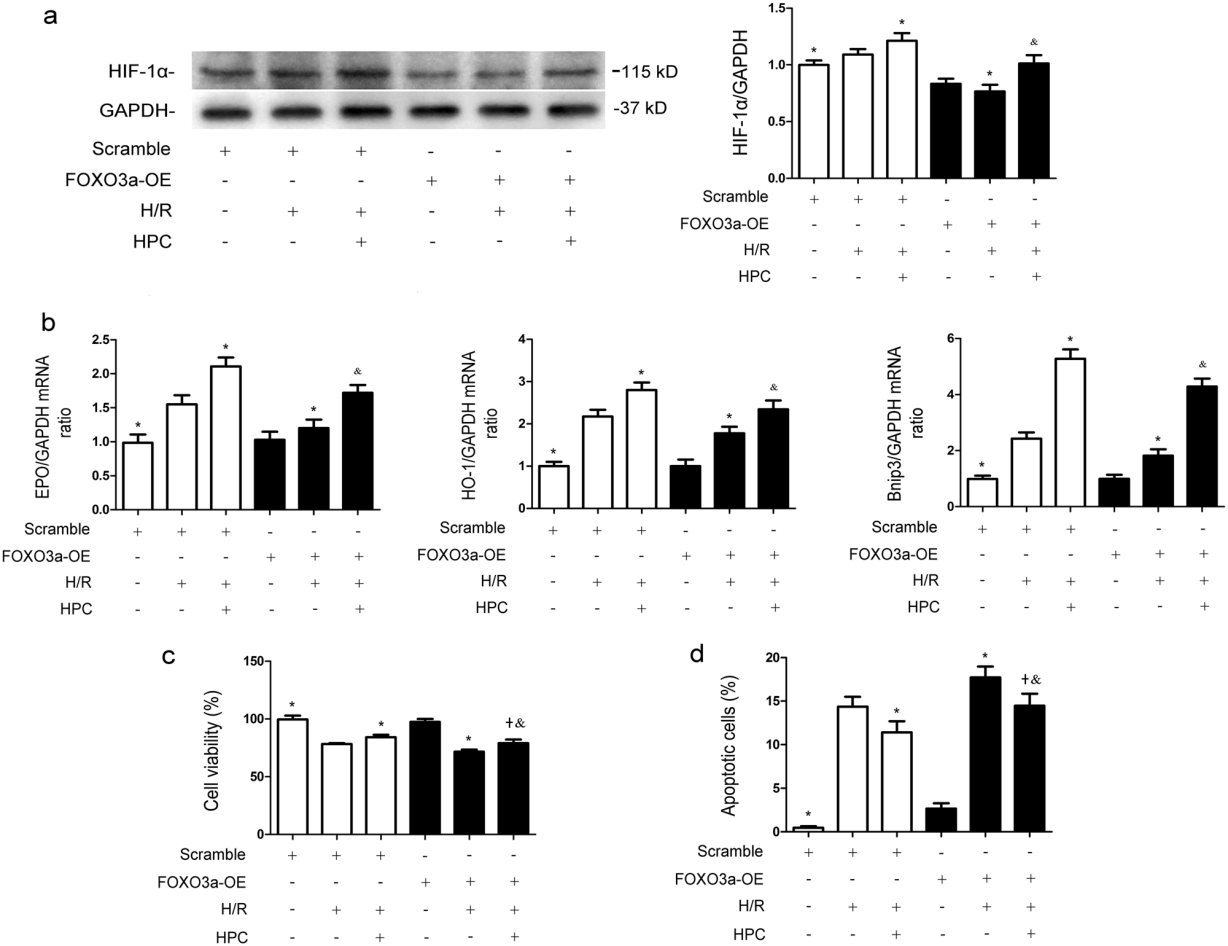
The level of FOXO3a affects the expression of HIF-1α protein and HPC-conferred renoprotection during H/R injury in vitro. **a** Cells were transfected with pEX-3-FOXO3a. After 48 h, they were cultured under normal conditions or exposed to H/R injury with or without prior HPC. After reoxygenation for 2 h, cells were collected for western blotting detection of HIF-1α. **b** EPO, HO-1 and Bnip3 mRNA levels were measured by real-time qPCR. **c** Renal tubular cell viability was evaluated by CCK-8 assay. **d** Flow cytometry was used to analyze the percentage of apoptotic cells. All the data are derived from an experiment that was repeated at least three times. The data are presented as the mean ± S.D. *n* = 3^*^.*P* < 0.05 vs. Scramble + H/R; ^&^*P* < 0.05 vs. Scramble + HPC + H/R; ^+^*P* < 0.05 vs. FOXO3a-OE + H/R

As shown in Fig. 3a, b, the levels of HIF-1α protein and mRNA of EPO, HO-1 and Bnip3 were all increased after H/R injury (*P* < 0.05 vs. Scramble); HPC significantly increased their expressions after H/R injury (*P* < 0.05 vs. Scramble + H/R). Overexpression of FOXO3a reduced HIF-1α protein expression and EPO, HO-1, Bnip3 mRNA levels in the HPC + H/R group (*P* < 0.05 vs. Scramble + HPC + H/R), indicating a negative effect of FOXO3a on the HIF-1α expression and its transcriptional function during HPC-mediated renal protection against H/R injury.CCK-8 and flow cytometric analysis showed that FOXO3a overexpression with pEX-3-FOXO3a before H/R exposure significantly attenuated HPC-conferred promotion of cell viability (*P* < 0.05 vs. Scramble + HPC + H/R) (Fig. 3c) and reduction of apoptotic cells (*P* < 0.05 vs. Scramble + HPC + H/R) (Fig. 3d), suggesting that FOXO3a may alleviate HPC-induced protection through reducing HIF-1α during renal H/R injury.

### SGK1 mediates the protective effect of hypoxic preconditioning during renal hypoxia/reoxygenation injury by promoting autophagic flux in vitro

**Fig. 4:**
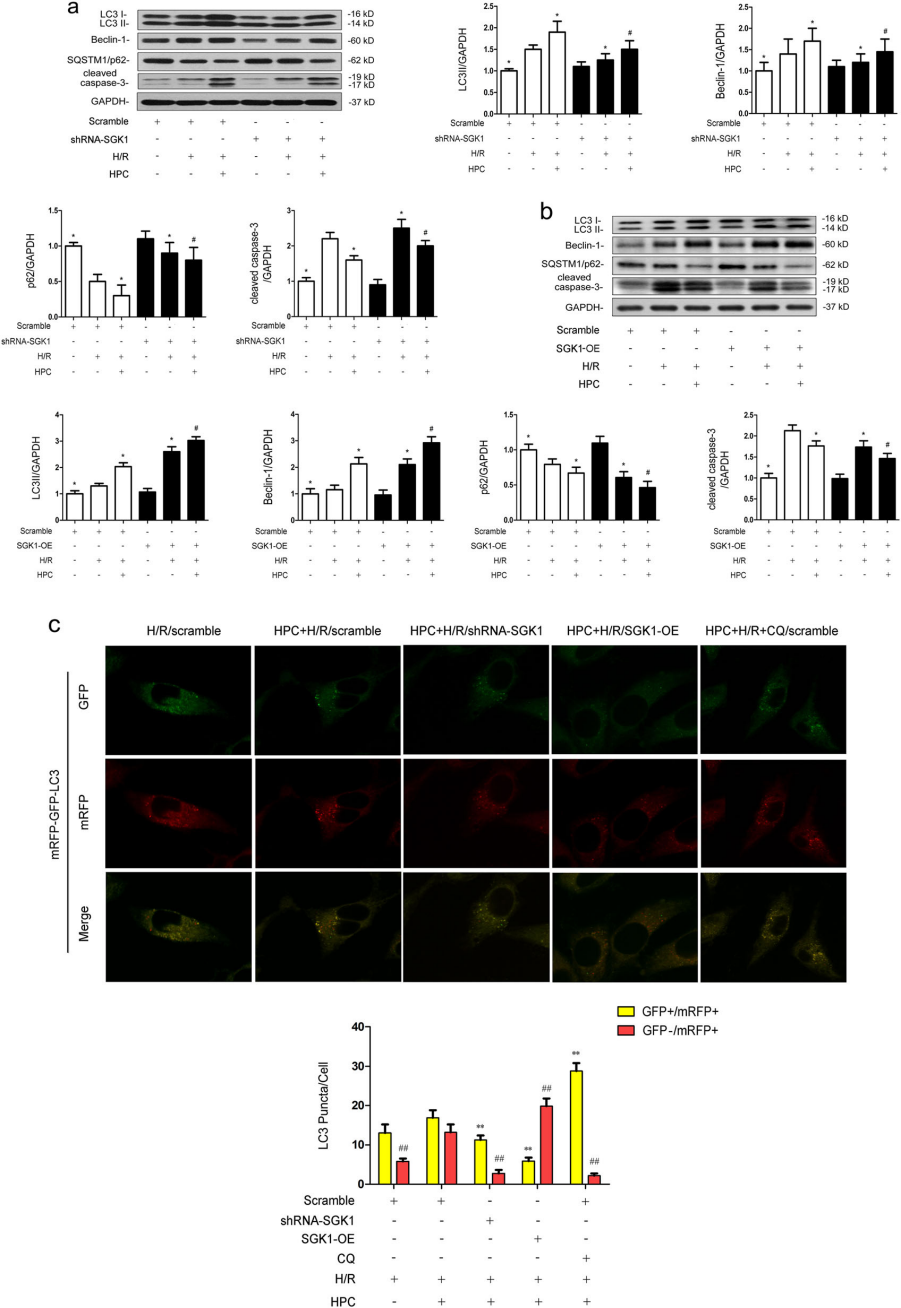
HPC-mediated autophagy induction during renal H/R in vitro depends on SGK1. **a**, **b** Cells were transfected with Scramble, shRNA-SGK1 or pEX-3-SGK1. After 48 h, they were cultured under normal conditions or exposed to H/R injury with or without prior HPC. After reoxygenation for 2 h, cells were collected for western blotting detection of LC3II, Beclin-1, SQSTM/p62, and cleaved caspase-3. **c** HK-2 cells were firstly infected with Scramble, shRNA-SGK1 or pEX-3-SGK1, and then infected with adenovirus encoding mRFP-GFP-LC3. After 12 h cells were exposed to H/R injury with or without prior HPC. After reoxygenation for 2 h, the cells were collected and observed under a confocal microscope. The data are derived from an experiment that was repeated at least three times and are presented as the mean ± S.D. *n* = 3^*^.*P* < 0.05 vs. Scramble + H/R; ^#^*P* < 0.05 vs. Scramble + HPC + H/R; ^**^P < 0.05^##^,*P* < 0.05 vs. Scramble + HPC + H/R

Pretreatment with HPC increased LC3II, Beclin-1 levels, and decreased SQSTM1/p62 expression after H/R injury (*P* < 0.05 vs. Scramble + H/R) (Fig. 4a, b). However, LC3II and Beclin-1 protein levels were decreased, and SQSTM1/p62 level was increased in HK-2 cells after shRNA-SGK1 transfection (*P* < 0.05 vs. Scramble + HPC + H/R) (Fig. 4a). Conversely, LC3II and Beclin-1 levels increased and SQSTM1/p62 level decreased in the SGK1-OE + HPC + H/R group (*P* < 0.05 vs. Scramble + HPC + H/R) (Fig. 4b). We also detected that the level of cleaved caspase 3 protein was increased in HPC-pretreated cells after transfected with shRNA-SGK1 (*P* < 0.05 vs. Scramble + HPC + H/R) (Fig. 4a), which was reversed in HPC-pretreated cells transfected with pEX-3-SGK1 (*P* < 0.05 vs. Scramble + HPC + H/R) (Fig. 4b). The immunoblotting analysis of autophagy-related proteins suggests that the promotion of autophagic flux by HPC is mediated by SGK1.

Cells were infected with the tandem red fluorescent protein (RFP)-green fluorescent protein (GFP)-LC3 adenovirus to monitor dynamic changes in autophagic flux. The GFP signal is quenched in the acidic or proteolytic conditions of the lysosome lumen, but RFP is relatively stable; therefore, colocalization of GFP and RFP indicates an autophagosome that has not fused with a lysosome (colocalized green and red dots in merged images appear yellow), while autolysosomes are labeled red. As shown in Fig. 4c, pretreatment with HPC increased the autolysosome population in H/R-exposed cells, which was counteracted by shRNA-SGK1 transfectionas indicated by decrease of autophagosomes and autolysosomes (*P* < 0.05 vs. Scramble + HPC + H/R). Overexpression of SGK1 accelerated autophagic activity obviously as manifested by decrease of autophagosomes and increase of autolysosomes (*P* < 0.05 vs. Scramble + HPC + H/R). Moreover, addition with chloroquine (CQ) before HPC significantly increased the number of autophagosomes in the HK-2 cells (*P* < 0.05 vs. Scramble + HPC + H/R). These results validate that SGK1 promotes autophagic flux by HPC during renal H/R injury rather than blocking its degradation pathway.

### Ischemic preconditioning induces autophagy and alleviates ischemia/reperfusion-induced renal damage in vivo

**Fig. 5:**
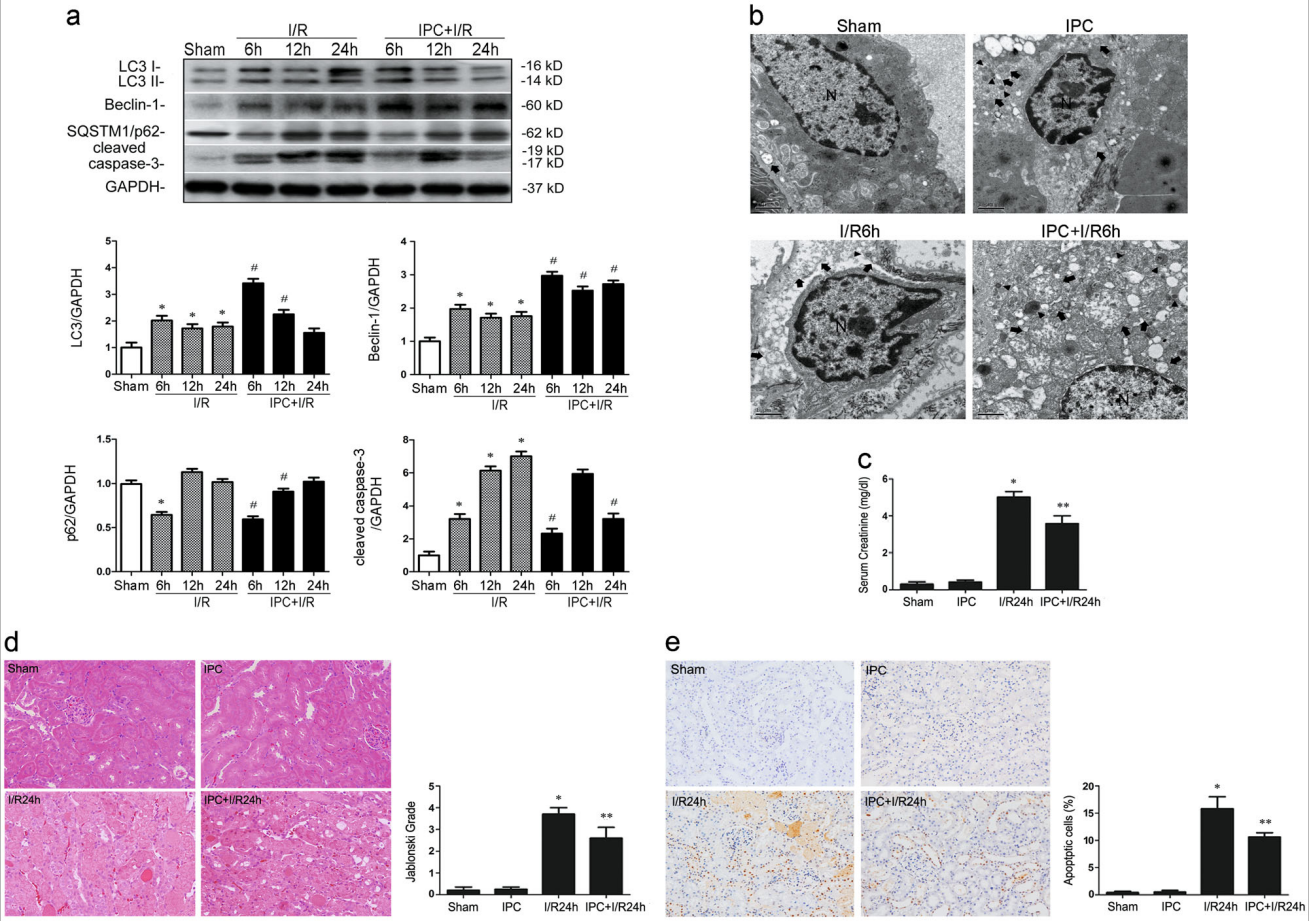
Ischemic preconditioning (IPC) induces autophagy and has a protective effect in an renal ischemia/reperfusion (I/R) model in vivo. **a** Male Sprague–Dawley (SD) rats were subjected to sham operation, single IPC, or 40-min ischemia followed by 2-h, 6-h, 12-h or 24-h reperfusion with or without prior IPC, which consisted of four cycles of 8 min of clamping the left renal artery separated by 5 min of reperfusion. Western blotting and subsequent data analysis were performed to detect LC3II, Beclin-1, SQSTM1/p62 and cleaved caspase-3 protein levels. Representative data are presented. **b** Intracellular ultrastructural features of autophagy were observed by transmission electron microscopy (TEM). N: nucleus; arrowhead: double-membraned autophagosome; arrow: lysosome. Magnification, ×20,000; scale bar = 1 μm. **c** Serum creatinine (Scr): renal function parameter. Representative images of renal histology and quantitative analysis of tubular injury and apoptosis are presented. Hematoxylin-eosin (H&E) staining (**d**) and terminal deoxynucleotidyl transferase-mediated nick-end labeling (TUNEL) staining (**e**) of kidney sections. Representative slides from each group are presented. Magnification, ×200. All the data are derived from an experiment that was repeated at least three times and are presented as the mean ± S.D. n = 5^*^.*P* < 0.05 vs. Sham; ^#^*P* < 0.05 vs. I/R group at the corresponding reperfusion time; ^**^P < 0.05 vs. I/R-24 h

As shown in Fig. 5a, LC3II and Beclin-1 expressions were increased in all I/R groups compared with the Sham groups, reaching a peak at I/R-6 h (*P* < 0.05 vs. Sham). Along with these changes, SQSTM1/p62 levels reached the nadir at I/R-6 h, which presented opposite change with the extension of reperfusion time. IPC significantly upregulated Beclin-1 protein expression at 6 h, 12 h, and 24 h after reperfusion (IPC + I/R-6 h vs. I/R-6 h; IPC + I/R-12 h vs. I/R-12 h; IPC + I/R-24 h vs. I/R-24 h; *P* < 0.05), and increased LC3II expression and SQSTM1/p62 degradation at I/R-6 h and I/R-12 h (IPC + I/R-6 h vs. I/R-6 h; IPC + I/R-12 h vs. I/R-12 h; *P* < 0.05). Moreover, IPC decreased cleaved caspase-3 at I/R-6 h and I/R-24 h (IPC + I/R-6 h vs. I/R-6 h; IPC + I/R-24 h vs. I/R-24 h; *P* < 0.05). Following this discovery, transmission electron microscopy (TEM) was used to detect morphological alterations associated with autophagy in renal tissues at I/R-6 h, at which point most of the autophagy-related proteins were expressed. The data showed that intervening with simple IPC did not change the number of autophagic vesicles in the kidney but did significantly increase them in the I/R-6 h group (*P* < 0.05 vs. I/R-6 h) (Fig. 5b). An analysis of renal function parameters showed decreased serum creatinine (Scr) in IPC-pretreated rats compared with the corresponding I/R group (IPC + I/R-24 h vs. I/R-24 h; *P* < 0.05) (Fig. 5c). Furthermore, H&E staining of renal tissues showed a wide range of cell necrosis, loss of the brush border, cast formation, increased tubule dilatation and degeneration of tubular cells at I/R-24 h (*P* < 0.05 vs. Sham) (Fig. 5d). IPC significantly alleviated the pathological changes (*P* < 0.05 vs. I/R-24 h). In addition, TUNEL assays confirmed there was less apoptosis in the IPC + I/R-24 h group than the I/R-24 h group (*P* < 0.05 vs. I/R-24 h) (Fig. 5e).

### Ischemic preconditioning affects SGK1-mediated signaling in a time-dependent manner during renal ischemia/reperfusion injury in vivo

**Fig. 6:**
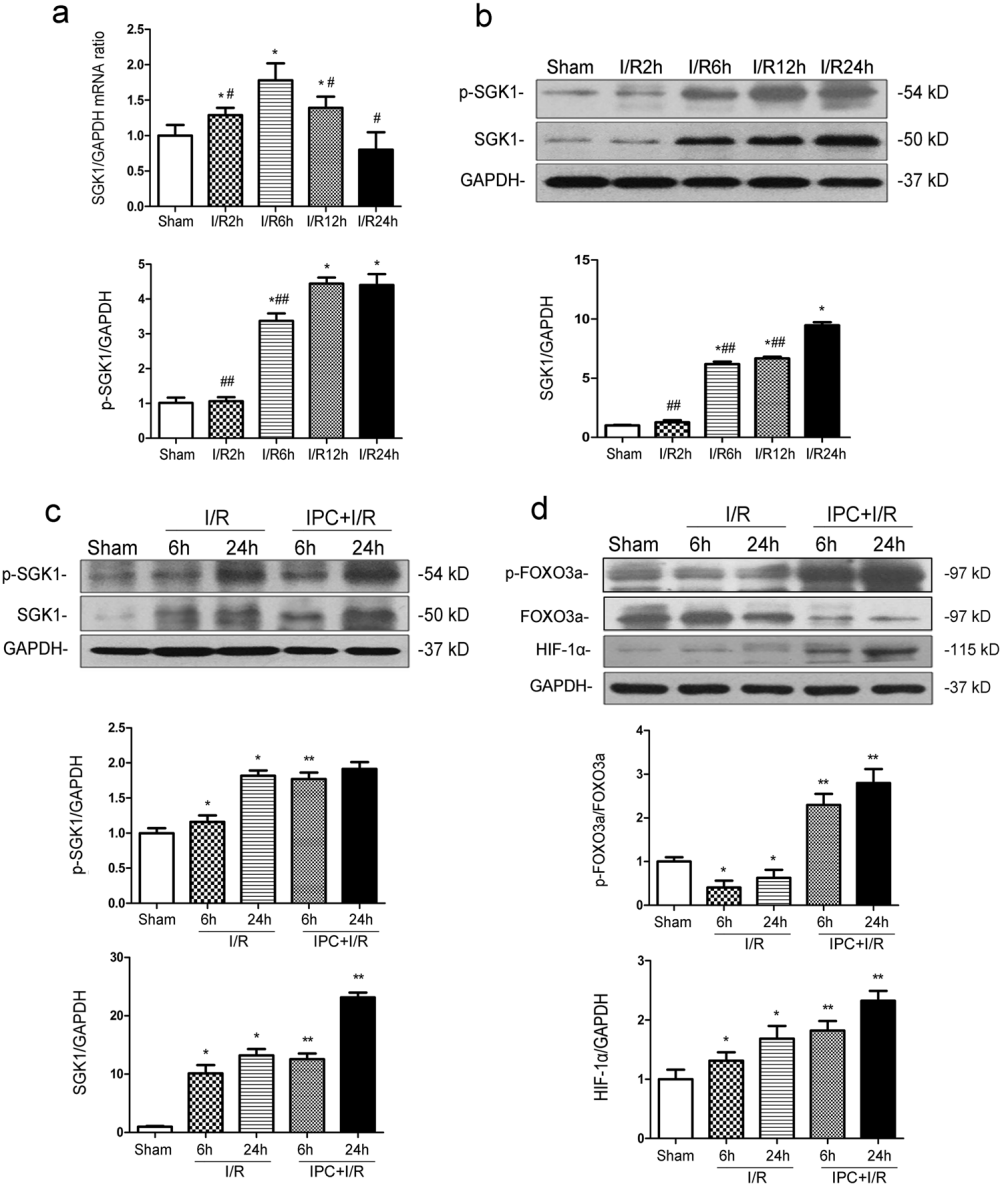
Ischemic preconditioning (IPC) changes SGK1/FOXO3a/HIF-1α protein expression during renal I/R injury. Male Sprague-Dawley (SD) rats were subjected to sham operation, single IPC, or 40-min ischemia followed by 2-h, 6-h, 12-h and 24-h reperfusion with or without prior IPC, which consisted of four cycles of 8 min of clamping the left renal artery separated by 5 min of reperfusion. **a** SGK1 mRNA levels in renal tissues from I/R-injured rats were measured by real-time qPCR and normalized to GAPDH expression. **b** Western blotting and subsequent data analysis were performed to detect p-SGK1 and SGK1 protein levels in renal tissues from I/R-injured rats. **c**, **d** Western blotting and data analysis were performed to detect p-SGK1, SGK1, p-FOXO3a, FOXO3a and HIF-1α expression levels in renal tissues from I/R-injured rats with or without prior IPC. Representative data are presented. All the data are derived from an experiment that was repeated at least three times and are presented as the mean ± S.D. *n* = 5 ^*^.*P* < 0.05 vs. Sham; ^#^*P* < 0.05 vs. I/R-6 h; ^##^*P* < 0.05 vs. I/R-24 h; ^**^P < 0.05 vs. I/R group at the corresponding reperfusion time

Although SGK1 mRNA levels began decreasing after I/R-6 h (Fig. 6a), we observed a gradual increase of SGK1 and p-SGK1 protein levels in renal tissues from I/R-injured rats compared with the sham-treated ones as the reperfusion time lengthened (*P* < 0.05 vs. Sham) (Fig. 6b). Western blotting showed that IPC significantly increased SGK1, p-FOXO3a/FOXO3a and HIF-1α protein expressions at I/R-6 h and I/R-24 h (IPC + I/R-6 h vs. I/R-6 h; IPC + I/R-24 h vs. I/R-24 h; *P* < 0.05) (Fig. 6c, d) but had little effect on p-SGK1 at 24 h. These data indicate a role of IPC in regulating the SGK1/FOXO3a/HIF-1α signaling pathway.

## Discussion

In recent years, IPC has emerged as an effective strategy for reducing renal I/R injury^4^. IPC triggers a number of autacoids (such as adenosine, bradykinin and opioids) that subsequently act on a complex intracellular signaling network; the reperfusion injury salvage kinase (RISK) and survivor activating factor enhancement (SAFE) pathways are two pivotal kinase signaling pathways mediating the beneficial effect of IPC^4,18^. Moreover, in early-phase protection by IPC, these pathways mainly converge on preventing the opening of the specific mitochondrial permeability transition pore (mPTP) by inhibiting GSK-3β^4,18^, whereas late-phase protection is dependent on the regulation of a series of key signaling molecules, such as reactive oxygen species, PKC, Nrf2, and, HIF^4,8,19–21^. Recently, IPC was demonstrated to induce autophagy^22,23^, which plays a critical role in protecting against I/R injury in the kidney and other organs^1,2^. However, the understanding of autophagy in IPC-induced protection, as well as its molecular mechanism in renal I/R injury, is limited.

In accordance with earlier observations providing convincing evidence for a protective role of IPC, we confirmed in this study that IPC protected both cultured HK-2 cells and rat kidney tissue against hypoxic and ischemic injury, as evidenced by increased cell viability and a lower rate of apoptosis in vitro and decreased Scr levels and renal structural damage in vivo. The present study showed that IPC activated autophagy, as indicated by a significant increase in Beclin-1 and LC3II protein levels, a decrease in SQSTM/p62, and an increase in autophagic flux. The TEM analysis revealed an increased number of autophagic vacuoles in IPC-treated rats. To further determine the role of autophagy in IPC-induced renoprotection, HK-2 cells were treated with 3-MA prior to H/R injury. We demonstrated that 3-MA treatment prevented IPC-induced autophagy and alleviated the protection afforded by IPC. Thus, these results confirm that the protective effects conferred by IPC may be mediated by the upregulation of autophagy in response to I/R injury.

As a part of the RISK pathway, Akt (also called PKB) plays a pivotal role in defending many organs against I/R injury^24^. Inhibiting Akt prevents the renoprotection afforded by acute IPC^3^. SGK1 is a serine/threonine protein kinase that is 45–55% homologous to Akt, and both kinases can be activated by PI3K, which is known to promote cell survival in response to stress stimuli^25^. Rusai et al.^26^ reported that erythropoietin (EPO) promotes SGK1 activation and downregulation of SGK1 ameliorated the anti-apoptotic effects of EPO during renal I/R injury, which indicates that SGK1 might facilitate the beneficial effects of EPO under ischemic conditions. However, studies investigating whether SGK1 is also involved in the beneficial effect of IPC are mostly lacking.

In this study, we found for the first time that IPC upregulated both the expression and phosphorylation of SGK1 both in vivo and in vitro. To investigate whether the induction and activation of SGK1 have significant impacts on the protective effect of IPC, we inhibited SGK1 expression in vitro using shRNA. We showed that SGK1 knockdown aggravated H/R-induced cell damage and weakened the protective effect of HPC against H/R injury. SGK1 overexpression alleviated H/R–mediated cell injury and exaggerated HPC-afforded protection. These results reveal the protective role of SGK1 against H/R injury and its involvement in HPC-induced protection in HK-2 cells. Consistently, SGK1 has also been shown to counteract apoptosis following H/R in vitro and ischemia in vivo in other organs. Previous studies showed that SGK1 knockdown aggravates H/R-induced cardiomyocyte damage^27,28^. Furthermore, SGK1 overexpression reduced apoptosis in rat hippocampal neurons subjected to I/R injury both in vivo and in vitro^29^. However, here SGK1 knockdown did not abrogate the protection conferred by HPC, which indicates that IPC may act via additional SGK1-independent mechanisms to mediate renoprotection.

Notably, we found that the effect of SGK1 on autophagy parallels its renoprotective activity. Moreover, inhibiting SGK1 significantly downregulated autophagy and enhancing SGK1promoted autophagy, suggesting that SGK1 is involved in the IPC-mediated upregulation of autophagy and thereby plays a protective role in IPC against H/R injury.

Autophagy regulatory mechanisms have been extensively investigated^30^. To the best of our knowledge, there are two classical autophagy regulatory mechanisms. Activation of AMPK (AMP-activated protein kinase) in response to low cellular nutrient status inhibits mTOR (mammalian target of rapamycin) and activates ULK1 (unc51-like autophagy activating kinase 1) via phosphorylation^6^. Another canonical autophagy regulatory pathway is HIF-1α dependent. HIF-1α upregulates its targets Bnip3 and Bnip3L and thereby mediates mitochondrial autophagy, which functions as a protective stress response by removing harmful and dysfunctional mitochondria and thus preventing the activation of apoptosis^31,32^.

Emerging evidence suggests that the PHD/HIF oxygen-sensing pathway is required for the IPC-induced protection against I/R injury^33–35^. IPC upregulates cardiac HIF-1α, and partial deficiency of HIF-1α abrogates IPC-induced protection against myocardial ischemia^22^. Xu et al.^36^ reported that IPC-mediated HIF-1α stabilization upregulates miR-21 in the kidney, which decreases the translation of programmed cell death protein 4 (PDCD4) and thereby inhibits apoptosis. While previous studies have demonstrated that HIF-1α is required for IPC-induced protection against I/R injury, none have explored the relationship between IPC-induced HIF-1α stabilization and autophagy.

In this study, we found that IPC significantly increased HIF-1α protein level, while SGK1 overexpression increased and SGK1 knockdown decreased HIF-1α levels. Based on the findings detailed above and those from this study, we suggest that IPC increases renal cell tolerance to I/R injury via HIF-1α-dependent autophagy regulated by SGK1 activation.

Autophagy has been described as an evolutionarily conserved pathway that mediates cellular degradation through lysosome pathways. Autophagy itself is paradoxical in that it can both promote cell survival and induce cell death, depending on the environmental stressors, which include changes in oxygen availability^37^. Specifically, mild levels of autophagy could constitute a protective mechanism by recycling damaged cellular components under conditions of moderate hypoxia stress, while excessive autophagy induced by severe prolonged hypoxia may lead to programmed cell death, also known as autophagic cell death. Furthermore, pro-survival autophagy upon exposure to short-term hypoxia is mediated by a HIF-1α-dependent pathway, while pro-death autophagy in response to prolonged hypoxia is predominantly dependent on AMPK/mTOR^37,38^. We posit that HIF-1α activation, in a manner mimicking IPC, is a promising target for pharmacological preconditioning that deserves further investigation.

FOXO3a, as a member of the forkhead transcription factor family, has been reported to actively promote apoptosis by inducing Bcl-2 family members^39^. Fujiki et al.^28^ showed that phosphorylation of FOXO3a is associated with increased translocation of FOXO3a from the nucleus to the cytoplasm, which inhibits FOXO3a-dependent nuclear transcription and promotes HK-2 cell survival. SGK1 acts as a strong anti-apoptotic kinase by phosphorylating FOXO3a and suppressing FOXO3a-mediated transcription^16^. FOXO3 is implicated in the integration of metabolic status. While the mammalian heart is exposed to starvation in which cardiomyocytes are subjected to metabolic stress, FOXO3a phosphorylation is decreased through AMPK activation, which thereby inhibits insulin-like growth factor 1 (IGF-1) and induces cell cycle withdrawal^40^. Moreover, a brief preconditioning stimulus affords neuroprotection through promoting phosphorylation of FOXO3a and GSK-3β^41^. Recent studies indicated that FOXO3a activation can not only decrease the transcriptional activity of HIF-1, but also result in a rapid increase in HIF-1α degradation through the lysosomal and proteasomal proteolysis pathways^13,42^. To further investigate the mechanism of HIF-1α stabilization by SGK1, we examined the effects of IPC on FOXO3a phosphorylation in kidney. We found that IPC markedly increased FOXO3a phosphorylation both in vivo and in vitro, which is consistent to the former study^41^, and this effect was suppressed by SGK1 inhibition and enhanced by SGK1 overexpreesion in vitro. Moreover, our study showed that FOXO3a overexpression not only decreased HIF-1α protein levels but also inhibited HIF-1α transcriptional activity, as evidenced by decreased mRNA levels of the HIF-1 target genes EPO, HO-1, and Bnip3 known to regulate autophagy^21,43^. Our previous study showed that DMOG induced delayed renal protection against I/R injury in mice, which were alleviated by inhibition of inducible nitric oxide synthase (iNOS). It is likely that iNOS could be induced by HPC in this study. The specific role of iNOS on autophagy during IPC deserves further study^8^.Considering all the data, we conclude that HPC may promote the stabilization of HIF-1α during H/R in a SGK1/FOXO3a-dependent manner, thereby attenuating H/R-induced injury. However, HIF activation has also been reported to enhance cell death via the induction of proapoptotic targets, especially under conditions of severe (anoxia) or prolonged hypoxia. The previous study reported that FOXO3a transcript levels accumulate in an HIF-1-dependent way and inhibit HIF-1-mediated apoptosis when exposed to severe hypoxia^44^. Thus, FOXO3a and HIF-1 may act as components of a concerted cellular response to hypoxic stress. FOXO3 and HIF-1 may have significant synergistic effects that help to amplify their individual beneficial effects and overcome their deficiencies. Future studies are needed to clarify the crosstalk between the two pathways.

In conclusion, this study indicates that IPC ameliorates renal I/R injury by promoting autophagy and exerts renoprotection via the SGK1 signaling pathway. We further provide a new insight into autophagy regulatory mechanisms. Notably, the functional analysis of SGK1 was performed only in vitro in this study; in vivo genetic models will be required for a thorough understanding of the role of SGK1 in IPC and its regulation of autophagy. Nevertheless, these findings suggest that SGK1 is a possible regulator of IPC-induced autophagy, and the induction of appropriate autophagy is a potential and promising therapeutic strategy for protecting against AKI.

## Materials and methods

### Animal experiments

Male Sprague-Dawley rats (230–270 g) were provided by the Super-B&K Laboratory Animal Center (Shanghai, China). Rats were housed in a temperature-and humidity-controlled environment with a 12:12 h light–dark cycle and were allowed free access to standard rodent chow and tap water. The use of rats in this study was approved by the Institutional Animal Care and Use Committees of Fudan University. Rats were anesthetized with sodium pentobarbital (50 mg/kg ip) and were kept on a heating pad to maintain their body temperature at 37 °C. Rat models of warm renal ischemia/reperfusion injury were established by clamping the left renal arteries for 40 min after right nephrectomy. For the IPC protocol, rats were subjected to four cycles of 8-min left renal artery ischemia separated by 5-min reperfusion periods before 40 min of left renal ischemia, as described previously^45^. Reperfusion was initiated by removing the clamp for the indicated time. Sham-operated animals underwent similar operative procedures without occlusion of the left renal artery.

### Cell culture and treatment

Human proximal tubular epithelial cells (HK-2) were purchased from American Type Culture Collection (ATCC, Manassas, VA, USA) and cultured in Dulbecco’s modified Eagle’s medium (DMEM; ThermoFisher, Hudson, NH, USA) supplemented with 10% heat-inactivated fetal bovine serum (FBS; ThermoFisher, Hudson, NH, USA). Modified oxygen and glucose deprivation (OGD) was used as a model of in vitro ischemia conditions. OGD of HK-2 cells was induced by changing the medium to serum/glucose free DMEM medium and then incubating them in a Modular Incubator Chamber (MIC-101; Billups-Rothenberg, Del Mar, CA, USA), and the chamber was flushed with 94% N_2_ and5% CO_2_ to maintain the oxygen concentration at 1%. After 15 h of hypoxia, the medium was replaced with fresh oxygenated medium containing serum and glucose, and the cells were returned to normoxic conditions for 30 min, 2 h or 6 h. For the hypoxic preconditioning protocol, cells were subjected to OGD for 6 h, followed by 2 h of reoxygenation before prolonged H/R injury. Cells in the normoxic group were cultured in a normoxic chamber (21%O_2_) for the indicated time periods. To suppress autophagy, 5 mM 3-methyladenine (3-MA; Sigma-Aldrich, St. Louis, MO, USA) was added to the medium 1 h before each experiment.

### Lentiviral particles and construct vector transfection

A recombinant lentivirus vector pGLV3 harboring a short hairpin RNA sequence targeting SGK1 (pGLV3-shRNA-SGK1) and the negative control (pGLV3-null) were produced by GenePharma (Shanghai, China). The target sequence of SGK1 shRNA is 5′- GGCAGAAGAAGTGTTCTATGC-3′. The complete sequences of SGK1 and FOXO3a mRNA including both the 5′ and 3′ UTRs were previously submitted to GenBank (Homo sapiens SGK1, NM_005627.3; Homo sapiensFOXO3, NM_001455.3).The expression vectors pEX-3 (PGCMV/MCS/Neo)-Control, pEX-3-SGK1 (SGK1 overexpression) and pEX-3-FOXO3a (FOXO3a overexpression) were purchased from or constructed by GenePharma (Shanghai, China). Cells were transfected with lentiviral particles or construct vector for 48 h and then incubated with fresh medium.

### Renal function detection

The renal function of post-ischemic kidneys was assessed by measuring serum blood urea nitrogen (BUN) and creatinine (Cr) levels. The samples were measured using a HITACHI 7180 automatic biochemical analyzer (Hitachi, Shanghai, China).

### Histological examination

Paraffin-embedded kidney sections (4 μm) were stained with H&E. Morphological assessments were performed by an experienced renal pathologist who was blinded to treatment group. More than 10 high-power fields (×400) including both the cortex and outer medulla were randomly selected. Tubular injury was graded in terms of tubular dilatation, tubular cell necrosis, debris accumulation, and cast formation on a scale from 0 to 4, as outlined by Jablonski et al.^46^. Higher scores indicate more severe damage: 0, none; 1, <5%; 2, 5–25%; 3, 25–75%; and 4, >75%.

### Transmission electron microscopy

The kidney was rapidly removed, and a specimen of therenal cortico-medullary boundary zone was sectioned into 1 × 1 × 1 mm^3^ fragments at 4 °C. The tissue block was fixed in 2.5% glutaraldehyde overnight at 4 °C. After a wash in phosphate buffer, the fixed tissue was post-fixed in 1% osmium tetroxide for 1 h at 4 °C. The specimen was dehydrated and embedded, and ultrathin sections were stained with uranyl acetate and lead citrate. A pathologist blinded to the study protocol analyzed the samples using a transmission electron microscope (Tenai G2 Spirit; Hillsboro, OR, USA).

### Autophagy flux detection

The cells were infected with adenovirus encoding mRFP-GFP-LC3 (Hanbio, Shanghai, China). Twelve hours after adenovirus transduction, the cells were subjected to different interventions as described above. The cells were then visualized using a confocal microscope (Olympus, Tokyo, Japan). The number of autophagosomes (red and green dots) and autolyosomes (red-only dots) per cell was calculated, and autophagic activity was evaluated using Image-Pro Plus software (Media Cybernetics Inc., Rockville, MD, USA). In total 20 μM Chloroquine (Sellect, Houston, TX, USA) was added to the medium 1 h before each experiment for autophagy suppression.

### Cell viability assay

Cell viability was detected with a Cell Counting Kit-8 (CCK-8, Dojindo, Kumamoto, Japan) according to the manufacturer’s instructions. The absorbance was measured using a microplate reader at a wavelength of 450 nm. The percentage of living cells was calculated based on the ratio of absorbance of the experimental group to that of the normoxic group.

### LDH release assay

The extent of cell death was quantitatively assessed by measuring the release of lactic dehydrogenase (LDH) into the culture media using the Cytoxicity LDH assay Kit-WST (Dojindo, Kumamoto, Japan) according to the manufacturer’s protocol. The absorbance of each sample was read at 490 nm using a microplate reader.

### Annexin V-FITC/PI staining and TUNEL assay

The cells were collected and incubated with 100 μl of 1 × binding buffer containing 5 μl of Annexin V-FITC and 5 μl of PI (ThermoFisher, Hudson, NH, USA). After a 30-min incubation in the dark at room temperature, the cells were analyzed by flow cytometry (BD FACSCalibur™, Becton Dickinson, NJ, USA). Annexin V^+^/PI^−^ cells were considered early apoptotic cells. Apoptotic morphology was detected by TUNEL assay according to the manufacturer’s instructions (Roche Diagnostics, Mannheim, Germany).The number of TUNEL-positive cells was evaluated in 10 fields per section and five sections per kidney.

### Quantitative RT-PCR

Total RNA was extracted from cells (2 × 10^6^) and rat renal tissues (0.1 mg) using TRIzol (ThermoFisher, Hudson, NH, USA) according to the manufacturer’s protocol. Aliquots (5 mg) of RNA were reverse transcribed to cDNA using the Superscribe First-Strand Synthesis System (ThermoFisher, Hudson, NH, USA). Primers for RT-qPCR used here are shown as follows: SGK1 forward 5′-CTCTTTCCAGACTGCTGACAAACT-3′, reverse 5′-AACGATGTTTAGGGAGTGCAGATA-3′; EPO forward 5′-TCACTGTCCCAGACACCAAA-3′, reverse 5′-CACTGACGGCTTTATCCACA-3′; HO-1 forward 5′-GCCAGCAACAAAGTGCAAGA-3′, reverse 5′-GTGTAAGGACCCATCGGAGAA-3′; Bnip3 forward 5′-GCCATCGGATTGGGGATCTAT-3′, reverse 5′-GCCACCCCAGGATCTAACAG-3′; GAPDH forward 5′-ATGGGGAAGGTGAAGGTCG-3′, reverse 5′-GGGGTCATTGATGGCAACAATA-3′. qRT-PCR was conducted according to the manufacturer’s instructions (ThermoFisher, Hudson, NH, USA). Reactions were run on a real-time PCR system (ABI PRISM 7700; Applied Biosystems, Foster, CA). Gene expression was detected with the SYBR Green RT-PCR Kit (ThermoFisher, Hudson, NH, USA), and relative gene expression was determined by normalization to GAPDH and the 2^-ΔΔCT^ method.

### Immunoblot analysis and antibodies

Whole cell or frozen kidney tissue lysates were extracted. The protein concentration was measured using the DC Protein Assay Kit (Bio-Rad Laboratories, Shanghai, China). Equal amounts of protein were separated on SDS-polyacrylamide gels and transferred onto nitrocellulose membranes. The membranes were then blotted overnight at 4 °C with antibodies against SGK1, phospho-SGK1, FOXO3α, phospho-FOXO3α, Beclin 1, and cleaved caspase-3(1:1000; Cell Signaling, Danvers, MA, USA); HIF-1α and LC3B (1:500; Novus, Littleton, CO, USA); SQSTM1/p62 (1:1000, Abcam, Shanghai, China); and GAPDH (1:5000; Sigma-Aldrich, St. Louis, MO, USA). After three washes with Tris-buffered saline containing 0.05% Tween-20 (TBST) for 15 min, the membranes were incubated with secondary goat anti-rabbit or goat anti-mouse IgG antibody (1:5000, Jackson, West Grove, PA, USA) for 1 h. The protein bands were visualized using ECL Plus (Amersham) according to the manufacturer’s instructions and developed on film.

### Statistical analyses

All values are presented as the mean ± S.D. Statistical analysis was conducted using GraphPad Prism 5.0. Group differences were analyzed by one-way ANOVA followed by Turkey’s post-test. *P* < 0.05 was considered statistically significant.
